# Study of Effectiveness of Prior Knowledge for Smart Home Kit Installation

**DOI:** 10.3390/s20216145

**Published:** 2020-10-29

**Authors:** Yang Hu, Diane J. Cook, Matthew E. Taylor

**Affiliations:** 1 The School of Electrical Engineering and Computer Science, Washington State University, Pullman, WA 99163, USA ; djcook@wsu.edu (D.J.C.); mtaylor3@ualberta.ca (M.E.T.); 2 Department of Computing Science, University of Alberta, Edmonton, AB T6G 2R3, Canada; 3 Alberta Machine Intelligence Institute, Edmonton, AB T5J 3B1, Canada

**Keywords:** smart home kit installation, prior knowledge, online tutoring system

## Abstract

Smart-Home in a Box (SHiB) is a ubiquitous system that intends to improve older adults’ life quality. SHiB requires self-installation before use. Our previous study found that it is not easy for seniors to install SHiB correctly. SHiB CBLE is a computer-based learning environment that is designed to help individuals install a SHiB kit. This article presents an experiment examining how smart home sensor installation was affected by knowledge gained from two methods, SHiB CBLE, and a written document. Results show that participants who were trained by the CBLE took significantly (p<0.05) less time in the installation session than those in the control group. The accuracy rate of SHiB kit installation is 78% for the group trained by the CBLE and 77% for the control group. Participants trained by the CBLE showed significantly (p<0.01) higher confidence in the actual installation than those in the control group. These results suggest that having a training before the actual installation will help installers avoid unnecessary work, shorten the installation time, and increase installers’ confidence.

## 1. Introduction

A report regarding baby boomers [1] reveals that, by 2035, one in five people in the United States will be aged 65 or older. Most older adults prefer to “age in place”, which means to stay in their own houses for as long as possible. Unfortunately, aging brings many challenges for older adults due to their cognitive decline, chronic diseases, and limited physical activity. Daily routine changes are considered signs of possible risks or diagnosis of diseases. Activity patterns obtained from daily routines can be the base for such predictions. Studies of ubiquitous systems regarding improving the life qualities of older adults are prevalent.

Ubiquitous computing has been extended to various computer science research areas as far back as Mark Weiser [2], when he proposed the paradigm in the 1990 s. Data are typically collected passively and can be applied to many areas, such as context-awareness by recognizing body sounds [3], indoor localization [4], residential routines [5], context-aware smart environments [6], security monitoring [7], and so on. Researchers developed ubiquitous technologies to improve life quality for older adults. Personal health devices [8] can detect patients’ abnormal status. The context-aware real-time assistant (CARA) [9] can provide personalized health-care services for older adults. Behavior analysis system [10] can predict anomalous behaviors of older adults and disabilities. Persuasive technology [11] can motivate older adults to adopt healthy lifestyle habits, such as doing exercise. Adults’ frailty can even be detected [12] by smartphones that have an accelerometer.

Gerontechnology is an interdisciplinary field combining gerontology and ubiquitous computing, aiming to allow senior participants to live independently for longer and improve their quality of life. It often focuses on matching a living environment to senior adults’ health, housing, mobility, communication, leisure, and work needs [13]. The Smart-Home in a Box (SHiB), which was developed by the Washington State University Center for Advanced Studies in Adaptive Systems (CASAS)[14], is a kit containing all the components of a smart home, designed to be installed by non-expert users. The goal of the SHiB, once installed, is to recognize senior individuals’ activities by comparing their current activities with past routines.

SHiB is a kit containing motion sensors, area sensors, temperature sensors, relays, and an Ethernet server. Motion sensors and area sensors are passive infrared (PIR) sensors. Motion sensors are downward-facing units with a view that is limited to a 4’ × 4’ region directly below. At the same time, area sensors have a field of view covering most of an entire room. The sensor message field contains an arbitrary string. Outputs of PIR sensors are binary state values of either “ON” or “OFF”, Messages transferred from temperature sensors are a number value or a description of their state in this field [14,15,16,17]. Sensor locations are critical because because accurately labeled data is necessary for activity recognition systems. Each sensor has a unique label that corresponds to its collected data. For instance, if a motion sensor is labeled as a “dining table” sensor but is placed above a reading chair, then the data will be incorrectly treated as a training dataset for daily diet routine recognition [18]. The project’s long-term purpose is to produce a smart home kit that can be easily installed, and then use it to collect large amounts of data related to user activity for research purposes.

Our prior study of the usability [18] of the smart home kit by senior participants (average age 69.3) indicates that knowledge about the installation is essential to a successful installation. The study indicates that the installation instructions are easy to understand for people with no engineering background. However, the actual installation process is confusing, particularly in where to install sensors. Therefore, the research suggests a need for an online pre-installation tutorial to provide participants with context before the installation process begins.

A website-based learning system, the Smart-Home in a Box Computer-based learning environment (SHiB CBLE) [19], was developed to address the shortcomings identified in the previous study [18]. The learning effectiveness of SHiB CBLE [19] indicates that there is no significant difference between elderly adults (≥55) and younger adults (<55) on post-test performance in placing sensors at the right location in the virtual learning environment. The tutoring system is accessible via the Internet (http://shib-test.byethost5.com/pre-tests/layout/layout.php?MID=publictry&GROUP=3), making it convenient for SHiB kit installers to have training before the actual installation.

Many studies have been conducted comparing learning effectiveness between computer-based teaching systems and traditional methods. However, a meta-analysis [20] indicates that most of the systems focus on higher education or K-12 populations. Hanid et al. [21] conducted a meta-analysis on learning effectiveness of virtual reality (VR) systems. The study indicates that most VR teaching systems focus on teaching STEM subjects, or professional subjects, such as nursing or computer programming. Most studies assess subjects’ performance via knowledge examination. A few studies examined practical exercises in the actual environment. Butt et al. [22] conducted a study on comparing the effectiveness of catheter insertion skill acquisition between nursing students trained by a VR learning system and traditional ways. The study examined students’ insertion skills in feedback from real patients. The results indicate that there is no significant difference in performance. However, students trained by the VR system took significantly a longer time in the training stage but spent significantly less time completing an insertion procedure. The current study focus on the non-technical population and the teaching content is directed toward physical installations of smart home sensors with written guidance.

This article reports a study’s results to evaluate the effectiveness of our existing (static) training instructions and the new SHiB CBLE. The study had two groups: an experimental group and a control group. Participants assigned to receive training in the SHiB CBLE were in the experimental group. Participants who were given a booklet having three worked examples were in the control group. Before the study, we developed three hypotheses:

1. Participants’ performance of the real installation in the experimental group will be better than those in the control group.
2. Participants in the experimental group will spend less time in the real installation than those in the control group.
3. Participants in the experimental group will be less confused about the installation than those in the control group.

The experiment design is described in the Methods section, the experiment results and relevant discussions are in the Results section, and the conclusions and suggestions for the future work that extends from the current study are in the Discussion section.

## 2. Methods

Knowledge about the SHiB installation can come from multiple sources: an observation of a demonstration, an oral description of the procedure, training in a virtual environment, or a brief introduction booklet. In this study, regardless of participants’ previous experience, two ways of teaching are compared. One method is through the online learning environment, SHiB CBLE , which has three training exercises and four post-tests. The other method is a booklet containing three examples of placing sensors in a layout. Participants in both groups were given installation instructions, which has been included in the SHiB kit for two years and was used by over eighty participants. The examples in the booklets are identical to the three training exercises in the SHiB CBLE to have participants in the two groups have the same information. The installation of the SHiB kit is in an empty on-campus apartment. The floor plan of the apartment is unknown to participants before they begin the physical installation.

### 2.1. Participants

Participants were enrolled from the Pullman, Washington area. Most of them are students from Washington State University. Twenty participants took part in the study. Twelve of them were males, and 8 of them were females. The participants’ age ranged from 18 to 55 years old, the mean value is 29.40, and the standard deviation is 11.06. Eight participants were married, 12 participants were single. Ten participants were Caucasian, four were Asian, two were Native American, 1 was African-American, and three did not answer this question. Twelve participants spoke English as their native language. Native languages spoken by the other 8 participants include Chinese, Hindi, Spanish, and others. The lowest level of education was completing high school, and the highest education level obtained was Ph.D. Each participant is identified as a two digit number. Participants voluntarily joined in this study. Participants were assigned randomly to two groups; each group had 10 participants.

### 2.2. Procedure

**Figure 1:**
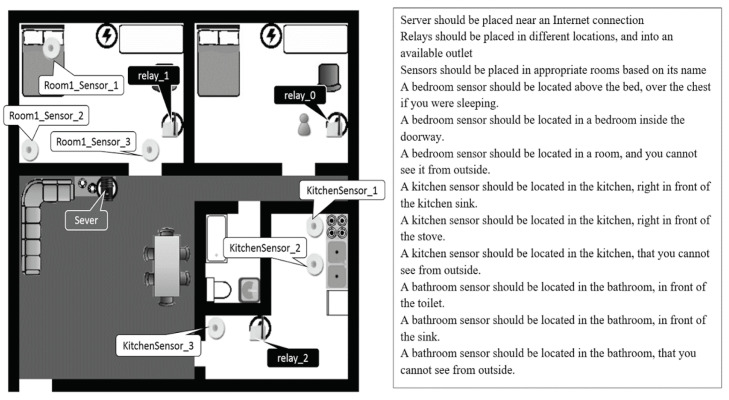
An example of a correct sensor placement for an apartment layout.

The experiment was conducted in an apartment with three bedrooms, one bathroom, one living room, and one kitchen for 1106 square feet. In the experiment, participants needed to install sensors in two bedrooms. Before beginning, each participant is surveyed about their background, including age, gender, native language, and others. In the experiment’s training part, participants in the control group were given a booklet with three examples of a correct smart home sensor arrangement on a floorplan layout. An example of the layout is shown in Figure 1. Details of the training contents of the control group are shown in Section 2.3.1.

**Figure 2:**
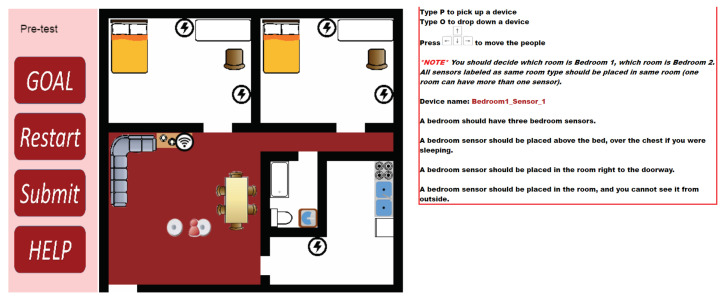
An example of SHiB CBLE user interface.

The researchers opened the SHiB CBLE in advance for the participants in the experimental group. The online training has a pre-test, three exercises in the training section, and four tests in the post-test section. The pre-test helps participants become familiar with the interface. Details of the training contents of the experimental group are stated in Section 2.3.2. Figure 2 shows an example of the interface of the SHiB CBLE.

During the experiment’s physical installation, each participant had a box containing 25 smart home devices. The devices include 14 motion sensors, two relays, two temperature sensors, one door-sensor, and one server. Among those devices, the training section exercises do not include exercises related to the temperature sensor and the door sensors. Besides the devices, each participant had a handbook with the details for installing each type of sensor. For instance, the motion sensors should be on the ceiling or at a high position on walls via adhesive strips. A specific port of the server should connect to the Ethernet cable. Relays should be on walls via adhesive strips near power plugs. The door sensor has two magnetic components: one should be on the door frame, and the other should be on the door. The two components should be aligned and be next to each other together when the door closed. The handbook contains general information about each type of sensor’s placement, which is a reminder for participants in the actual installations.

### 2.3. Training Contents

The researchers printed the teaching contents of the control group to a three-page booklet. The format of each page is the same as Figure 1. The experimental group participants took three training exercises and four post-tests from the SHiB CBLE. The contents of the training exercises are similar to those of the control group. However, participants need to learn how to find the right positions for devices. In the first and second training exercises, participants follow a worked example to place the devices. If a participant made an error, the CBLE would give them suggestions and ask them to fix the error. In the third training exercise, the content is the same as the third working example in the booklet. However, CBLE participants do not know the solution in advance—they had to discover it independently. The CBLE gives hints if there are errors.

Section 2.3.1 discusses the three worked examples printed in the three-page booklet. Section 2.3.2 discusses details of the CBLE and covers the third training exercise and the four post-tests.

#### 2.3.1. Control Group

**Figure 3:**
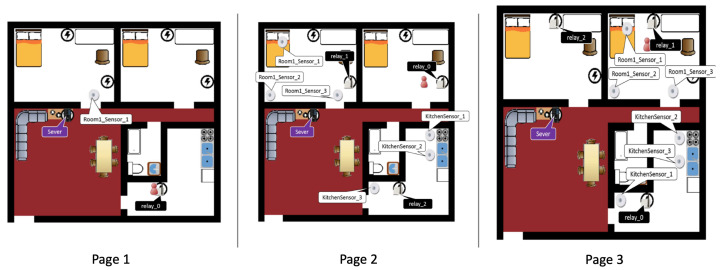
Worked examples in the control group’s instruction booklet.

Worked examples shown in the booklet are in a sequence from simple to complicated. Page 1 shows an example of a solution to place three devices. The problems on pages 2 and 3 are more complicated in which ten devices need to be placed. Example solutions on pages 2 and 3 show two ways for placing ten devices. The purpose of showing two solutions is to highlight alternate solutions to the learners. Figure 3 shows the three worked examples of the booklet. The house layouts of the three examples are identical. On page 1, the three devices are a room sensor, a relay, and a server. On pages 2 and 3, there are three room sensors, three relays, three kitchen sensors, and a server. The example on page 3 is an alternative solution to the problem set. For example, the three room sensors are placed in the bedroom on the left on page 2, while on page 3 they are placed in the right bedroom. Positions of relays and kitchen sensors on page 3 are changed from those on page 2.

#### 2.3.2. Experimental Group

**Figure 4:**
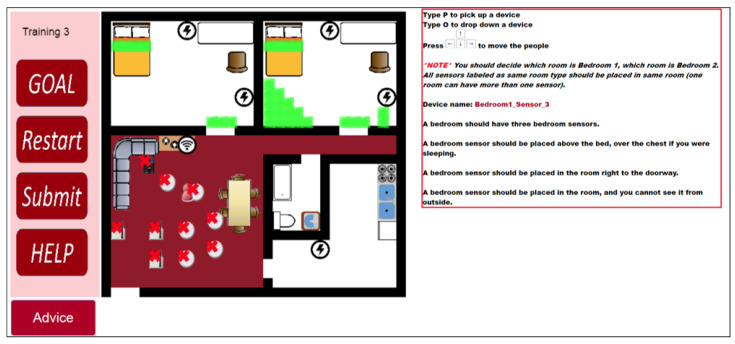
An example of hints for a room sensor.

**Figure 5:**
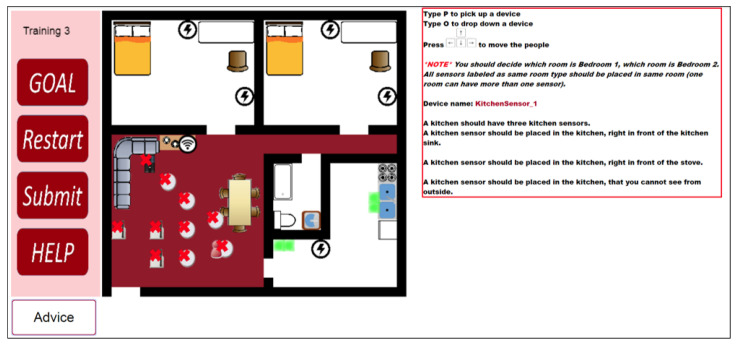
An example of hints for a kitchen sensor.

**Figure 6:**
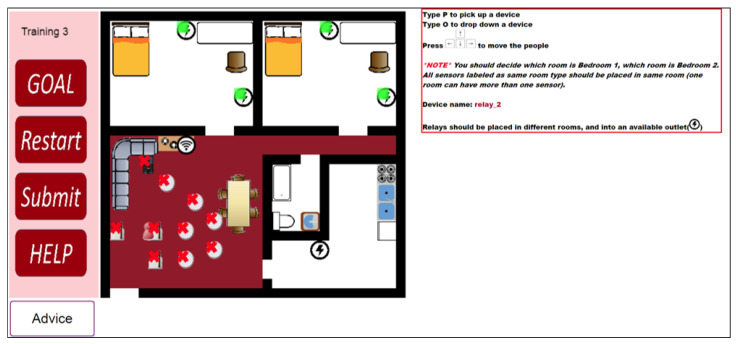
An example of hints for a relay

**Figure 7:**
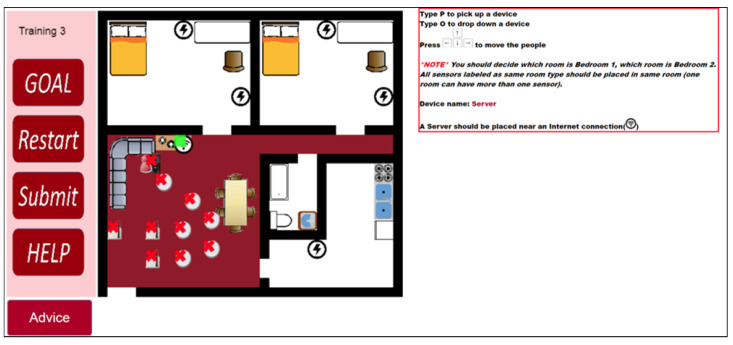
An example of hint for the server.

The room layouts for the three training exercises are identical to the contents of the control group booklet. In the first two training exercises, participants need to follow worked examples to place given devices. In the third training exercises, participants need to find the correct place for the devices. The tutoring system will give suggestions after participants placed all the devices. Figure 4, Figure 5, Figure 6 and Figure 7 are examples of the third training exercise. Every time a participant presses the “Submit” button, the CBLE verifies the devices’ placement. When the CBLE detects a device is at the wrong place, it shows an X on the device. The participant can move the red avatar and use it to pick up the incorrectly placed device and then click the “Advice” button at the bottom right. The CBLE will then highlight all the correct areas for that device. As shown in Figure 4, the name of the device is “Bedroom1_Sensor_3” All sensors applied in the study are manufactured by Card Access Engineering Inc, Draper, UT 84020, USA, a partner of Control4, which is in red at the right. The tutorial suggests three correct places: above the bed, at the door, or in the area in a bedroom such that no one can see it from outside the room. In the third exercise, the bedroom sensors’ location should also be different from that in the second exercise. Therefore, the suggested areas exclude those used in the second exercise. In this example, only two types of spots are highlighted in the left bedroom because the third type of spot in the second exercise was used, which is the area that no one can see from outside the bedroom.

Figure 5 shows a kitchen sensor example. The name of the example kitchen sensor is “KitchenSensor_1”, which is in red on the right. The example shows two places in the kitchen for the device: the spot in front of the sink and the corner behind the doorway to the kitchen. The tutorial suggests three types of spots for a kitchen sensor. They are at right under the sensor name. In this case, the spot in front of the oven is not highlighted, which indicates the participant placed the device on the spot in the second exercise.

In Figure 6 shows a relay placement example. As the tutorial suggests, relays should be close to outlets. Figure 6 highlights four outlets out of five. The learner used the one in the kitchen to place “relay_2” in the second training exercise.

Figure 7 shows a server placement example. The server should be close to the internet connection and the tutorial highlights it in the figure. Each layout has only one spot for a server as there is usually a single router in a house.

**Figure 8:**
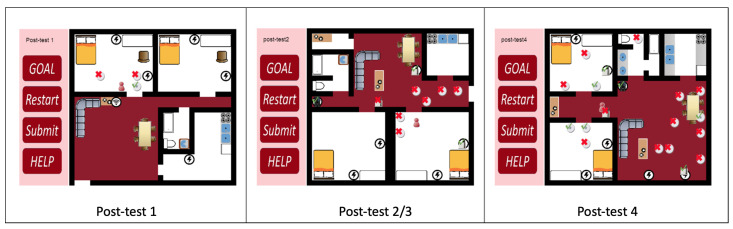
Examples of post-tests.

The four post-tests use three different house layouts. Post-test 1 tests three room sensors. Post-test 2 and post-test 3 use the same layout and both test six sensors, three relays, and a server. Post-test 3 requires an alternative solution from the solution submitted in post-test 2. Post-test 4 has a new layout, and it tests twelve sensors, three relays, and a server. Figure 8 shows examples of the post-tests. In post-tests, the CBLE verifies solutions if a participant wants to check her answers. The participant can continue to next test even if the CBLE detects errors.

### 2.4. Evaluation Metrics

**Figure 9:**
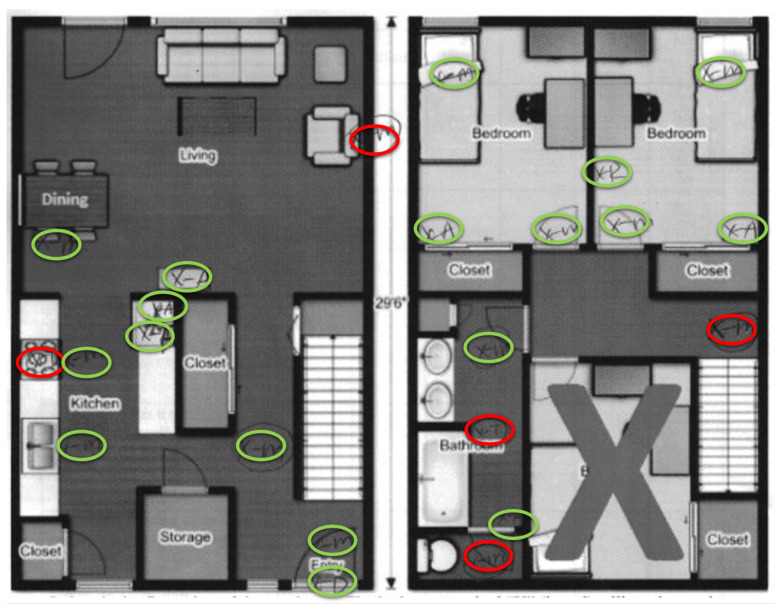
An example of evaluating an installation.

Participants’ performance depends on how accurately they installed sensors in the physical apartment. The researchers marked the spots that the participant has chosen to install each device. A spot is marked in the format of “x-a”, where “x” is the location and the letter “a” represents the initial letter of the sensor type. Particularly, “R” is the initial letter for relays, “M” is the initial letter for the motion sensor, “A” is the initial letter for area sensor, and “T” is the initial letter for the temperature sensor. Figure 9 shows an example. In the figure, researchers circled correctly placed devices in green and incorrectly placed devices in red.

Another evaluation metric is the time each participant spends in the experiment’s training section and installation section. We assume that having a better understanding of the installation requirements correlates with shorter installation time. We believe that the installation time is more important than the time spent training; shorter installation time saves senior residents’ energy and will help reduce the risk of indoor accidents. A decision change in the SHiB kit installation means the participant must reinstall a device, including climbing up and down a ladder (the risk of injury due to falling from ladders increases with age [23]). In contrast, time spent on training has minimal risk. After the entire installation, each participant took a survey. Their opinions about the installation were collected in the questionnaire and will become another metric for the experiment. Table 1 has the survey questions.

### 2.5. Data Analysis

We applied an Analysis of variance (ANOVA) analysis to our experiment data. To examine the validity of the ANOVA results, we applied the Shapiro–Wilk test for data normality evaluation and Bartlett’s test to evaluate the homogeneity of data variance. The Shapiro–Wilk test tests the null hypothesis that a sample population came from a normally distributed population and a p>0.05 indicates the null hypothesis is not rejected. Bartlett’s test tests the null hypothesis that there is no difference in variances between the groups. A p>0.05 indicates the null hypothesis is not rejected.

## 3. Results

The experiment results consist of three parts: the participants’ performance in the physical installation, their time cost in training and the physical installation, and their responses to the post-survey.

### 3.1. Performance of Installation

**Figure 10:**
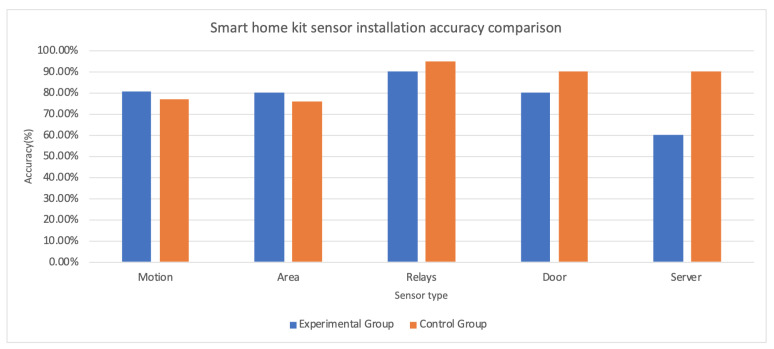
Sensor installation accuracy comparison.

This section presents the results of the experiment. Table 2 shows the results of the actual installation. Figure 10 presents comparisons of sensor installation accuracy in percentage. The experiment enrolled ten participants; each of them installed the devices once. Table 2 shows the types of devices, and the number of devices under each type is different. The table also records “failures”, which is the number of times that the device type was installed at an incorrect position. A server installation failed when it was not connected correctly to the Ethernet. The average accuracy rate represents the mean value of the number of correctly installed devices over the total number of devices in each group.

The physical installation performance results do not support the hypothesis (1), which is that the experimental group will perform better than the control group. The experimental group performed better at installing motion sensors and area sensors than the control group. The control group performed better in server installation than the experimental group. The accuracy rate of installing temperature sensors, relays, and door sensors are similar in the two groups. A reason for the high failure rate in installing the server in the experimental group is that the experimental group participants learned that the server should connect to the Ethernet. However, the training does not have instructions for how to connect to the Ethernet. The server has two adaptors, but only one of them works for the connection. Participants have to read the installation manual carefully to know which port to connect. Temperature sensor installation has specific requirements. An oven temperature sensor should be placed right above the oven—many participants placed the sensor on the ceiling, which was too far from the oven to be effective. Similarly, the bathroom temperature sensor was difficult to place—it had to be placed close enough to the shower so that it could detect hot water, but far enough that it did not become damp from steam.

### 3.2. Time Spent

The researchers timed how long it took for each participant to finish each section of the experiment. As the two training methods were designed to have different training times, the results reflect the training methods’ purpose. The mean time of control group participants spent in training is 6.4 min. In comparison, the mean time spent by the experimental group is 59.4 min. Shorter training time in the control group is due to the 3-page booklet’s brevity given to each participant. Each page has an intuitive layout with sensors marked at correct places. Participants can spend as much time as they want on the booklet. Most participants spent less than 10 min reading through the material. On the other hand, the SHiB CBLE training program has eight tasks in total. Each task requires the participants to work on each sensor in a virtual environment. The program is designed to provide 45 to 60 min of training for learners.

Table 3 shows the results of the time spent by each participant installing the SHiB. We conducted the Shapiro–Wilk test for data normality evaluation. The result (p>0.05) indicates that data comes from a normal distribution. We then conducted Bartlett’s test for data variance homogeneity evaluation. The result (p>0.05) indicates that the two groups’ time consumption data variance are equal. Because participants were randomly assigned to the two groups, participants’ time consumption results are independent. With the satisfaction of ANOVA assumptions, we then performed an ANOVA test on the installation time. The result implies that participants in the experimental group spent significantly (p<0.05) less time (with a mean of 64.2 min) than participants in the control group (with a mean of 80.6 min). The result supports the hypothesis (2) of the study. The result indicates that participants can spend less time installing the smart home kit in their own house if they had trained by the SHiB CBLE program instead of following static directions.

### 3.3. Post-Survey Results

Questions of the post-survey are in two categories: questions 1 through 6 relate to the ease of installing smart-home devices, and questions 7 through 10 relate to the training and overall installation. Each question’s value represents the degree of agreement to the question, “5” stands for strongly agree or very easy, while “1” stands for strongly disagree or very difficult, and “N/A” means that the participant did not answer that question. The analyses of the results do not include those responses with “N/A”. Questions are listed in Table 1.

Table 4 shows the results for the first six questions of the post-survey. We conducted the Shapiro–Wilk normality test to the averaged score. The result (p>0.05) indicates that the data comes from a normal distribution. We then conducted Bartlett’s test to the same data. The result (p>0.05) indicates the two groups’ averaged scores variance are equal. The participants were randomly assigned to the two groups, which satisfies the sample independence assumption for ANOVA. Therefore, the three assumptions of valid ANOVA are satisfied; we then performed an ANOVA to examine the differences between the two groups. The two groups’ average rating values for the first six questions imply that the experimental group’s responses are significantly more positive (p<0.01) than those from the control group. The six questions focus on participants’ subjective responses to the actual installation. Questions 1, 3, and 6 are designed to collect participants’ viewpoints of the ease of the physical sensor and server installations. Questions 2 and 4 are designed to examine participants’ viewpoints on how easy the installation manual and the sensors’ names are to understand. Question 5 is designed to evaluate participants’ opinions about the ease of installation for other people. The responses to the first six questions from both groups suggest that participants believe the physical installation would be easier for participants in the experimental group than those in the control group. This result implies that the experimental group participants had higher confidence in the actual installation than those in the control group.

Table 5 shows the results of questions 7 through 10. An ANOVA test is conducted on the average values of questions 7 through 10 in the post-survey to examine the significance between the two groups. The results show there is no significance between the two. Questions 7 and 10 evaluate how easy participants thought it was to understand the training material and how useful the training was for the physical installation. The results imply that training is easy (mean = 4.0) for the control group participants, but neutral (mean = 3.7) for those in the experimental group. However, participants in the experimental group think the training helps the installation (mean = 3.9), while the responses from participants in the control group are neutral (mean = 3.2). The length of the whole installation process is neutral for participants in both groups. Participants in both groups disagree with the statement that the installation was not frustrating.

Question 11 asks, “Would you recommend installing a Smart Home in a Box to others?” and was responded to by 18 out of 20 participants with “yes"; two participants did not answer this question. Question 12 asks “Did you receive any help with installing the sensors?”, answered with 6 “No” and 4 “Yes” in the experimental group, and 8 “No” and 2 “Yes” in the control group. Participants could receive some types of help in the installation; for example, researchers answered questions about the apartment floor plan or held the ladder while the participant was placing devices in high places. Researchers will not answer questions relating to the correct position for placing the devices.

Question 13 asks participants to comment on what part of the training seemed the most helpful. Table 6 shows the responses. “N/A” means the participant did not answer the question. Ten participants in the experimental group answered the question, while 8 participants in the control group left responses. In both groups, participants mentioned that the motion sensors’ training and the area sensors were helpful. Participants in the experimental group mentioned that the training for the relays’ position was helpful. In contrast, one participant in the control group mentioned the material for relays was confusing. Practicing on the SHiB CBLE was mentioned as helpful for participants in the experimental group. Participants in the experimental group also commented that the similarity between the SHiB CBLE and the actual installation was helpful.

## 4. Discussion

This article reports on a study that tests the effectiveness of teaching participants how to install smart home sensors. In the experiment, we compared how the installation process was affected by the two types of teaching methods. One uses a booklet with three floorplans associated with sensors marked at their correct positions. The other uses a computer-based learning environment, SHiB CBLE, which includes three training exercises and four post-tests. During the actual installation stage, subjects from the two groups have the same installation instructions, which contain all the information they need to complete the process. We explored and examined the effects of two different ways participants could become familiar with the smart home system installation via three hypotheses: (1) the accuracy rate of physical smart-home installation, (2) the time spent in the installation, and (3) participants’ subjective viewpoints about the installation.

Twenty participants took part in the experiment. They were randomly assigned to two groups: a control group and an experimental group; each group has 10 participants. Each participant received training at the beginning and then started the physical smart-home installation. The experiment was conducted in a rented apartment. After the installation, participants took a survey with a questionnaire about the installation. The authors evaluated the installation performance for each participant.

The experiment results show that the two groups’ accuracy rate is similar, 77% and 78%, with no significant difference. The results did not support our hypothesis (1), where we expected that the experimental group would perform better than the control group in placing sensors at the right positions. The results also differ from our previous study [19]. A major difference in the participants’ performance test stage, in the previous study, was that no extra guidance was given to participants. On the contrary, in the current study, installation instructions were available for both group participants.

Time consumption was significantly different between the two groups: time spent by participants in the experimental group is significantly (p<0.05) shorter than that in the control group. The results are consistent with a prior study [22] in that subjects trained by the learning system tend to spend less time in practical operations. The result suggests that training with the SHiB CBLE will help to reduce actual installation time, which implies reducing unnecessary work during the installation. That, in turn, could lower the risk of injury for installers, especially for individuals with mobility impairment.

The post-survey results imply that participants training with SHiB CBLE before the installation have significantly (p<0.01) more confidence than those training via a three pages booklet. Participants in the experimental group think that the SHiB CBLE helps them determine sensor positions, get familiar with the installation manual, and translate the content learned in the CBLE to the actual installation.

The current study results imply a possible application of computer-based learning systems in improving user experience in smart home sensor installation, particularly in reducing installation time and improving user confidence.

There are some limitations to this study. The sample size of participants is small when compared to the number of potential senior installers. Most of this study’s participants are young, with a mean age 29.4, and may not accurately represent a senior population. The SHiB CBLE helped reduce time spent in the actual installation, but needs improvement in increasing the actual installation’s accuracy rate. For example, details related to installing the door sensor and server should appear in the SHiB CBLE. A larger-scale experiment could examine the knowledge translation of the SHiB CBLE virtual environment to physical installations.

## Figures and Tables

**Table 1:** Post-survey questions.

Index	Question Content
1	How easy was it to install the sensors?
2	How easy was it to install the names and terms for items?
3	How easy was it to install the server box?	
4	How easy was it to follow the instruction manual?
5	How easy would it be for other people to install the sensors and server box?
6	Overall, how easy was the entire installation process?
7	How easy was it to follow the installation tutorial software?
8	Would you say that the installation process was long?
9	Would you say that you were frustrated or anxious while installing the sensors?
10	Would you say that the installation tutorial software was helpful for the actual installation?
11	Would you recommend installing a Smart Home in a Box to others?
12	Did you receive any help with installing the sensors?
13	What aspects of the installation tutorial software did you find most useful?

**Table 2:** Results of real installation.

	Motion		Area		Temperature		Relay		Door		Server		Average Accuracy
	Total	Failure	Total	Failure	Total	Failure	Total	Failure	Total	Failure	Total	Failure	Rate %
Experimental	140	27	50	10	20	10	20	2	10	2	10	4	
Accuracy		80.7%		80.0%		50.0%		90.0%		80.0%		60.0%	78.0%
Control	140	32	50	12	20	9	20	1	10	1	10	1	
Accuracy		77.1%		76.0%		55.0%		95.0%		90.0%		90.0%	77.6%

**Table 3:** Comparison of time spent in installation.

Control Group		Experimental Group	
Label No.	Installation (min)	Label No.	Installation (min)
2	105	1	73
3	101	4	56
5	70	6	84
8	55	7	58
9	76	10	75
12	79	11	67
13	108	14	61
15	90	16	40
17	67	19	61
18	55	20	67
mean(*)	80.6		64.2
min	55		40
max	108		84
std	18.7		11.5

(*)=p<0.05.

**Table 4:** Results of post-survey of questions 1–6.

Experimental Gorup							Control Gorup						
Participant No.	Q1	Q2	Q3	Q4	Q5	Q6	Participant No.	Q1	Q2	Q3	Q4	Q5	Q6
1	2	4	4	4	4	3	2	4	N/A	N/A	4	4	4
4	4	4	4	4	4	4	3	3	4	3	4	3	4
6	4	4	5	4	4	4	5	4	5	4	4	4	4
7	4	4	5	4	4	4	8	4	4	4	4	4	4
10	5	4	3	4	4	4	9	4	4	3	4	4	4
11	5	5	5	5	5	5	12	4	4	5	4	3	4
14	5	5	5	5	3	4	13	5	5	5	5	5	3
16	3	5	4	4	4	3	15	3	4	5	4	N/A	4
19	5	4	5	5	5	5	17	3	3	4	3	3	3
20	4	5	5	4	4	4	18	4	3	4	3	3	4
Average(**)	4.1	4.4	4.5	4.3	4.1	4.0		3.8	4.0	4.1	3.9	3.7	3.8

(**)=p<0.01.

**Table 5:** Post-survey results comparison on questions 7–10.

Experimental group					Control group				
Participant No.	Q7	Q8	Q9	Q10	Participant No.	Q7	Q8	Q9	Q10
1	4	5	4	5	2	N/A	2	2	4
4	4	5	2	5	3	4	3	2	N/A
6	4	3	2	4	5	N/A	2	2	N/A
7	4	4	3	5	8	4	3	1	1
10	3	3	2	3	9	N/A	3	3	N/A
11	4	2	1	3	12	N/A	4	2	4
14	4	2	2	2	13	N/A	3	3	N/A
16	4	3	3	4	15	3	5	4	3
19	3	2	2	4	17	4	3	4	4
20	3	4	2	4	18	5	3	2	N/A
Average	3.7	3.3	2.3	3.9		4.0	3.1	2.5	3.2

**Table 6:** Responses to Q13 of the experimental group and the control group.

Experimental Group		Control Group	
Participant No.	Response on Q13	Participant No.	Response on Q13
1	instructing every time and showing proper directions to set up the sensors	2	confused with relays
4	It help me understand where the ’area’ sensor should be placed	3	like enforce best places for sensors
6	place sensors and relays in the specific area	5	textbook is helpful for area sensors
7	the similarity between the software and manual instructions	8	textbook gives a general understanding of different sensor locations
10	knowing the placement of the sensors	9	N/A
11	locations of sensors	12	Floor plans with pictures is okay
14	Material is enough	13	N/A
16	practice, different steps	15	The figures showing positions of sensors might help
19	The visual displays translated over to the actual installation	17	all
20	going through the process of placing the items, the descriptions of how and where to place the items	18	The map (textbook) is useful

## Abbreviations

The following abbreviations are used in this manuscript.

SHiB	Smart Home in a Box
CBLE	Computer-based learning environment
PIR	Passive Infrared
ANOVA	Analysis of variance
